# Quality and stability of artemether-lumefantrine stored under ambient conditions in rural Mali

**DOI:** 10.1186/1475-2875-13-474

**Published:** 2014-12-04

**Authors:** John Gitua, Aaron Beck, John Rovers

**Affiliations:** College of Arts and Sciences, Drake University, 2507 University Avenue, Des Moines, IA 50311 USA; College of Pharmacy and Health Sciences, Drake University, 2507 University Avenue, Des Moines, IA 50311 USA

**Keywords:** Drug stability, Artemisinins, Infrared spectrophotometry, Magnetic resonance spectroscopy, Pharmacists

## Abstract

**Background:**

The quality and stability of anti-malarial drugs in the Global South has long been of significant concern. Drug quality can be affected by poor or fraudulent manufacturing processes, while drug stability is affected by temperature and humidity. Knowledge of drug quality and stability is often the unique contribution of pharmacists volunteering on short-term medical mission trips.

**Objective:**

To determine the quality and stability of artemether-lumefantrine 20/120 mg under ambient storage conditions in rural Mali.

**Methods:**

One unopened blister pack of artemether-lumefantrine 20/120 mg (IPCA Laboratories, Mumbai) was stored under ambient conditions in a warehouse in a Malian village for one year. A second pack from the same lot number was stored under temperature and humidity controlled conditions in a university laboratory. The active ingredients of tablets from both packages were analysed using thin layer chromatography, nuclear magnetic resonance and infrared spectroscopy. The IPCA samples were referenced for drug identity and content to an identical American made product (Coartem ®, Novartis Pharmaceuticals).

**Results:**

Thin layer chromatographs, nuclear magnetic resonance and infrared spectroscopy results were identical for both IPCA samples and the reference product.

**Conclusions:**

The IPCA products contained the same drugs in the same amount as on their package label and were identical to the reference product. It is concluded that they were of good quality. Spectroscopy results demonstrate the sample stored in Mali was stable for one year. Pharmacists volunteering on medical mission trips may dispense this product with confidence. At the end of a mission trip, pharmacists may store left over artemether-lumefantrine under ambient conditions for up to one year without concern for significant degradation of the active ingredients.

## Background

Drug quality in the Global South has long been recognized as an important problem. Pharmacists volunteering on medical mission groups are often the only members of the team who are trained in evaluating the quality and stability of the medications the team provides.

Nayyar *et al.* have classified poor quality drugs into three types [1]. Falsified drugs are fraudulently manufactured, with fake packaging and usually either contain no active ingredient or one other than that specified on the label. Substandard drugs result from poor manufacturing processes. They may contain too much or too little active ingredient, but are not manufactured with the intention to deceive. Degraded drugs were of good quality at the time of their manufacture, but have deteriorated since, often due to poor storage conditions. The first two classifications may be considered to be problems of genuinely poor quality, while the last classification is a problem of drug stability. Proper patient care requires any drugs dispensed to be both of good quality and adequate stability.

Given the hot and humid climate, drug stability and subsequent degradation would be expected to be an important problem in maintaining drug quality in sub-Saharan Africa. This may be especially problematic for anti-malarials [1]. Poor quality anti-malarials may result in underdosing patients and subsequently lead to treatment failure or drug resistance by the parasite [2].

A number of studies have evaluated anti-malarial drug quality and stability under conditions typically found in Africa. Studies used a variety of methods to determine drug quality and stability including storage under simulated tropical conditions, determining the amount of drug released after dissolution of a solid dosage form, chromatography and spectroscopy.

Ballereau *et al.* found that chloroquine stored for up to two years under ambient conditions in a clinic in rural Burkina Faso deteriorated by more than 10% by day 450 [3]. Risha *et al.* evaluated the drug release profile of four sulphadoxine-pyrimethamine (SP) and seven chloroquine products [4]. Two SP products failed to release ≥ 60% of sulphadoxine and ≥ 30% of pyrimethamine at baseline, three months and six months. By six months, two chloroquine products failed to release ≥ 80% of the drug. Using similar methods, Kayumba *et al.* tested three quinine and two SP products. By six months, one SP and one quinine product failed to release the required amount of drug [5].

Using high performance liquid chromatography (HPLC), Taylor *et al.* studied solid, oral liquid and parenteral dosage forms of a number of anti-malarials including various chloroquine and quinine salts, proguanil, and SP [6]. Between 4-38% of SP samples fell outside of British Pharmacopoeia (BP) standards. Most proguanil and quinine products met BP standards, while between 70-100% of chloroquine salts failed BP requirements. Poor quality assurance in the drug manufacturing process, rather than drug degradation, was ascribed as being the most likely cause of product failure.

Two studies evaluated the expiry date information on the labels of anti-malarial compounds in Kenya and Sierra Leone. An audit of anti-malarials purchased from 880 Kenyan retail outlets showed that over 90% of products were within their labelled expiration dates [7]. Amodiaquine products had the longest remaining shelf life (median 46 months) followed by SP (30 months), chloroquine (22 months), quinine (19 months) and artemisinin compounds (12 months). Sesay found that seven chloroquine products available in Sierra Leone had labelled shelf lives ranging from three to five years [8]. Although these studies did not explicitly evaluate drug stability, their labelled expiration information may be taken as a proxy measure of stability.

Several studies have evaluated the stability of artemisinin compounds. During stability studies as part of the commercial drug development process, Bardsley *et al.* noted that a chlorproguanil-dapsone-artesunate tablet showed an unknown degradation product after accelerated stability studies under simulated tropical conditions at 30°C and 65% relative humidity [9]. Using HPLC, HPLC-mass spectrometry (HPLC-MS), and nuclear magnetic resonance (NMR) spectroscopy, they identified the degradant as a product of a reaction between dapsone and either artesunate or succinic acid which is released when artesunate is exposed to moisture. Charrier *et al.* subjected artesunate-amodiaquine combination tablets to accelerated degradation at 50, 60, 70°C and 75% humidity [10]. Analysis by MS and NMR revealed an artesunate degradation product that was virtually undetectable in the non heat-stressed samples. Bate *et al.* obtained samples of artemether-lumefantrine that were 1 – 58 months past their labelled expiry date [11]. They found that 97% of expired products passed tests for tablet integrity and the presence and amount of active ingredients using thin layer chromatography, tablet disintegration and infrared (IR) spectroscopy but they did not study the samples using dissolution testing. They concluded that the usual two year labelled shelf life for stability could probably be safely extended.

Given the increasing drug resistance of *Plasmodium falciparum*, artemisinin combination therapy (ACT) has become the preferred treatment for malaria in sub-Saharan Africa [12]. A recent medical service trip to Mali provided an opportunity to evaluate the quality and stability of one ACT product.

Medicine for Mali is a volunteer organization that offers medical, public health, clean water and microfinance services to several villages in the Cercle of Kati, located in the Koulikoro Region (approximately 12°20’N by 8°20’W) southwest of the capital city, Bamako [13]. In February 2012, a team of volunteers working for Medicine for Mali provided health care services during a two week trip to the village of Nana Kenieba. Although the volunteers brought most of the necessary medications from the USA, ACT was supplied by the Malian government as artemether-lumefantrine 20/120 mg tablets (IPCA Laboratories, Mumbai).

### Objective

Medicine for Mali has worked in Nana Kenieba since 2000 and maintains a small warehouse in the village to store left over supplies between visits. The objective of this study was to determine the quality and stability of artemether-lumefantrine 20/120 mg tablets stored under ambient conditions in the warehouse for one year compared to identical tablets stored in a temperature and humidity controlled university chemistry laboratory for the same period of time as well as to an American made reference product.

## Methods

The day before the team returned to the USA from Mali, one unopened blister pack of 24 artemether-lumefantrine 20/120 mg tablets (IPCA Laboratories, Mumbai, Lot Number DY1571423, Expiry Date 6/2013) was placed in the warehouse in Nana Kenieba. The team brought a second identical, unopened package of artemether-lumefantrine 20/120 mg tablets from the same lot number back to the USA. These tablets were stored indoors in a temperature and humidity controlled university chemistry laboratory in the USA.

The tablets in the warehouse were stored under ambient conditions in a brick building that has a window and a roof but no means of controlling temperature or humidity. The climate in the Koulikoro Region ranges between an average daily temperature and rainfall of 21.4°C and zero respectively in January and an average temperature of 33.9°C in June and rainfall of 103.7mm in August [14]. The artemether-lumefantrine 20/120 mg tablets were stored under ambient conditions in the warehouse for one year and then shipped by commercial courier to a university chemistry laboratory for analysis in March 2013.

Coartem 20/120 ® (Novartis Pharmaceuticals Corporation, Suffern, New York, Lot Number F3060 Expiry Date 4/2015) was used as the reference product against which the identity and amount of the drugs in both IPCA samples were compared.

Two tablets for each drug sample were separately crushed to a fine powder using a mortar and pestle, transferred into a 125 mL Erlenmeyer flask and extracted three times using 20 mL of ethyl acetate as the extracting solvent. All three extracts for each sample were combined, dried using anhydrous sodium sulfate and then concentrated using a rotatory evaporator to give 0.263g of a yellow solid.

The melting point of the yellow solid was found to be in the range 80°C -120°C. The wide melting point range indicated that the extracts were a mixture of more than one component.

To confirm how many components were present in the yellow solid, thin layer chromatography (TLC) was used. TLC is a qualitative technique that uses the partitioning of the sample between a mobile solvent phase and a stationary phase. In this experiment, the stationary phase was silica gel 60 F_254_ plates. The mobile phase was prepared from 2 mL ethyl acetate, 1 mL glacial acetic acid and 9 mL toluene.

Further characterization of the components of the yellow extract was accomplished using two analytical techniques; nuclear magnetic resonance (NMR) and infrared (IR) spectroscopy. NMR spectroscopy is an analytical chemistry technique used to determine the content and purity of a sample as well as its molecular structure. NMR spectra obtained for both crude and purified drug samples were analysed using a Bruker Avance 400 MHz instrument followed by matching the obtained spectra with known spectral data as well as that of the standard. The analysis involved both the proton (^1^H) and isotope carbon (^13^C) experiments. In these experiments, the solid products were dissolved in deuterated chloroform (CDCl_3_), put in an NMR tube and then analysed using the NMR instrument at the respective radio frequencies.

Infrared (IR) spectra for both crude and purified drug samples were obtained using a Varian 2000 FTIR instrument which allows the analysis of neat liquid and solid samples without use of the more commonly used mineral oil (Nujol ®) for sample preparation. IR spectroscopy is a technique that identifies the different functional groups present on the structure of a given organic compound. If the functional groups have different types of bonds, they absorb the electromagnetic radiation at different wavelengths which are recorded on the IR spectrum as peaks. In comparing the sample IR spectrum to those in the spectral library, the identity of such functional groups can be confirmed.

## Results

TLC analysis of the developed plates under UV light at 254nm, showed one yellow spot attributed to lumefantrine (R_f_ = 0.19). A second spot attributed to artemether (R_f_ = 0.57) was observed when the plate was dipped into a methanolic sulfuric acid solution 5% and then dried on a hot plate.

The ^1^H NMR spectra for artemether displayed three distinct singlets at 5.29, 4.59 and 3.34 (OCH_3_) ppm while lumefantrine gave specific strong aromatic signals between 7.10 and 7.61 ppm, singlet at 5.22 and a broad singlet at 4.0 (OH) ppm.

The IR spectra showed the presence of artemether with the peaks at 2952, 1480, 1262, 1099, 872, 808 cm^-1^ and lumefantrine peaks at 3396 (OH group), 2928, 1584, 1484, 1083 cm^-1^.

From the analysis of the two chemical components of both the IPCA drugs stored under different conditions, and referenced to Coartem 20/120 ®, there was no difference in the amounts of artemether and lumefantrine between the three samples. The different laboratory analytical techniques used in this analysis exhibited consistency and reproducibility of the results since identical results were obtained using all analytical methods for both the samples of interest, as well as the reference product.

## Discussion

Drug quality and stability in the developing world is a serious concern. Although there have been some studies of artemisinin quality and stability [9–11], this study appears to be the first peer-reviewed study to both evaluate the products’ quality and then study its stability under ambient tropical conditions. The study products were also compared to a reference product. As such, this study answers two questions that pharmacists on medical mission trips are frequently concerned with. Is the product of good quality? Is it stable under these storage conditions? In both cases, these results suggest the answer is yes.

There is ample evidence that fake artemisinin compounds are common in Asia [15–18]. Although the setting for this study was Africa, the anti-malarials used to treat patients in the Medicine for Mali clinic were made in India and imported into Mali by the Malian government. Consequently, there is some risk that a fraudulent product could have been imported and patient care could have been compromised. These results demonstrate that the IPCA product was indistinguishable from a reference product made in the USA and was not a fraudulent one. As Malian officials continue to import Asian-made ACT for domestic use, the IPCA products evaluated in this study appear to be of good quality.

Experimental analysis of the drug samples revealed there was no difference in drug content between the products stored under ambient conditions in Mali or temperature and humidity controlled conditions in the USA compared to the reference product. This indicates that any drug degradation in the Malian warehouse was not different than any degradation seen in the product stored under temperature and humidity controlled conditions or the reference product.

Johnson *et al.* note that knowledge of drug storage and stability are a unique contribution of pharmacists to a medical mission team [19]. These authors make special note of the probability that there will be medication left over at the end of a short-term mission trip. Having a plan for disposition of left over medication is necessary.

In much of the developing world, safe disposal of medication is problematic since there are rarely Western style waste disposal systems. If medication cannot be appropriately disposed of, it poses a risk for both the environment and to local children who may find and consume left over medications. Ideally, any medication left at the end of a short-term mission trip should either be donated to a local medical clinic or stored safely until the next mission trip.

These results demonstrate that the artemether-lumefantrine compound studied was stable under ambient storage conditions in a high temperature and high humidity environment. This is consistent with the results of Bate *et al.* who found that even artemether-lumefantrine that was past its labelled expiry date was stable and not degraded [11]. Mission groups with access to a secure warehouse or other storage facility can consider storing this product for up to a year in the event they choose not to donate it to a local clinic.

## Abbreviations

ACT: Artemisinin combination therapy
BP: British Pharmacopoeia
HPLC: High performance liquid chromatography
IR: Infrared
MS: Mass spectrometry
NMR: Nuclear magnetic resonance
SP: Sulphadoxine-pyrimethamine
TLC: Thin layer chromatography.
